# Community- and hospital-acquired infections with oseltamivir- and peramivir-resistant influenza A(H1N1)pdm09 viruses during the 2015–2016 season in Japan

**DOI:** 10.1007/s11262-016-1396-9

**Published:** 2016-10-06

**Authors:** Akinobu Hibino, Hiroki Kondo, Hironori Masaki, Yoshinari Tanabe, Isamu Sato, Nobuhiro Takemae, Takehiko Saito, Hassan Zaraket, Reiko Saito

**Affiliations:** 1 Division of International Health, Graduate School of Medical and Dental Sciences, Niigata University, Niigata, 951-8510 Japan; 2 Masaki Respiratory Medicine Clinic, Nagasaki, Japan; 3 Infection Disease Control Section, Niigata University Medical and Dental Hospital, Niigata, Japan; 4 Yoiko-no-Syounika Sato Clinic, Niigata, Japan; 5 Division of Transboundary Animal Disease, National Institute of Animal Health, National Agriculture and Food Research Organization, Tsukuba, Japan; 6 Department Pathology, Immunology, and Microbiology, Faculty of Medicine, American University of Beirut, Beirut, Lebanon; 7 Center for Infectious Disease Research, Faculty of Medicine, American University of Beirut, Beirut, Lebanon

**Keywords:** Influenza virus A(H1N1)pdm09, Neuraminidase, Oseltamivir resistant, Antiviral drug susceptibility, Community transmission, Enzymatic property

## Abstract

We report five cases of community- and hospital-acquired infections with oseltamivir- and peramivir-resistant A(H1N1)pdm09 viruses possessing the neuraminidase (NA) H275Y mutation during January–February 2016 in Japan. One case was hospitalized and was receiving oseltamivir for prophylaxis. The remaining four cases were not taking antiviral drugs at the time of sampling. These cases were geographically distant and epidemiologically unrelated. The five viruses showed ~300-fold rise in IC_50_ values against oseltamivir and peramivir, defined as highly reduced inhibition according to the WHO definition. Overall, the prevalence of the H275Y A(H1N1)pdm09 viruses was 1.8 % (5/282). The resistant viruses possessed the V241I, N369 K, and N386 K substitutions in the NA that have been previously reported among A(H1N1)pdm09 to alter transmission fitness. Analysis of Michaelis constant (Km) revealed that two of the isolates had reduced NA affinity to MUNANA, while the other three isolates displayed a slightly decreased affinity compared to the sensitive viruses. Further studies are needed to monitor the community spread of resistant viruses and to assess their transmissibility.

**Electronic supplementary material:**

The online version of this article (doi:10.1007/s11262-016-1396-9) contains supplementary material, which is available to authorized users.

## Text

Neuraminidase inhibitors (NAIs), sialic acid analogues, are the drugs of choice for prevention and treatment of influenza [1]. NAIs block the viral neuraminidase (NA), an enzyme on the surface of the virus that is important for the release of newly formed virions from the host cells [2, 3]. Four NAIs (oseltamivir, zanamivir, laninamivir, and peramivir) are approved for influenza treatment in Japan [4]. Except for peramivir, NAIs are also licensed for prophylaxis against influenza [4]. Resistance to NAIs can be caused by a single mutation in the NA [2, 3]. The most frequent resistant-conferring mutation among the influenza A(H1N1)pdm09 is the H275Y. Viruses carrying this mutation possess cross resistance to oseltamivir and peramivir but remain susceptible to zanamivir and laninamivir [2, 3]. Community-acquired oseltamivir- and peramivir-resistant viruses with the H275Y mutation have been recently identified in several countries [5–7]. Community spread of resistant viruses raises concerns regarding their potential to replace sensitive strains as in the case of the resistant seasonal A(H1N1) viruses that spread globally between 2007 and 2008 [2, 3].

We report five cases of community- and hospital-acquired oseltamivir- and peramivir-resistant A(H1N1)pdm09 viruses during the 2015–2016 season in Japan.

In total, 664 nasopharyngeal swabs were collected from patients with influenza- like illness (ILI) who visited seven collaborative clinics or hospitals in Hokkaido, Niigata, Gunma, Kyoto Nagasaki and Okinawa, Japan between January 4 and February 29, 2016. Specimens were collected after rapid influenza antigen detection test (RDT) was positive for influenza A or B. Informed consent was obtained from the patients or their guardians along with demographic and clinical data. The Niigata University Ethics Committee has approved the study. Samples were sent from the medical facilities to Niigata University for further virological analysis. Clinical specimens were inoculated onto Madin-Darby canine kidney cells [8]. Samples with a positive cytopathic effect were typed and subtyped by using a cycling probe real-time PCR method which can identify influenza A(H1N1)pdm09, A(H3N2), B/Victoria, and B/Yamagata [8]. The assay can simultaneously detect the presence of the H275Y mutation in A(H1N1)pdm09 viruses [9]. A total of 282 influenza A(H1N1)pdm09, 1 A(H3N2), 25 B/Victoria, and 26 B/Yamagata lineage viruses were identified during the study period. Five of A(H1N1)pdm09 isolates (5/282, 1.8 %) were found to possess the H275Y mutation. This incidence is similar to the rate (1.0 %) reported by National Institute of Infectious Disease, Tokyo, Japan during 2015–2016 season (as of 4 March, 2016) [10]. However, this incidence was lower than that reported during the 2013–2014 season (4.1 %) [7].

One case of infection with H275Y mutant virus was detected in a hospitalized patient, while the other four were from patients presenting at community clinics.

Case 1. A 75-year-old woman was admitted to a hospital in Niigata City with relapse of multiple myeloma on January 13, 2016 (Table 1). Prior to infection, her roommate was diagnosed as influenza A by a RDT. The roommate started treatment with oseltamivir on January 21 and was discharged from the hospital on January 22. As a result, case 1 was administered a prophylactic dose of oseltamivir at 75 mg/day as of January 21. The patient developed cough on January 22, and fever (38 °C) on January 25. On the day of fever onset, an RDT was positive for influenza A and her treatment was changed to 100 mg peramivir. A reduced dose (adult dose is normally 300 mg) was given to the patient because she had a mildly impaired renal function as a complication to multiple myeloma (BUN 28 mg/dl, Creatinine 1.02 mg/dl). She received one dose of peramivir each on January 25 and 26 and her fever dropped to below 37.5 °C in the evening of the 26th. The influenza sample obtained on January 25 was found to possess the H275Y mutation by real-time PCR.

**Table 1:** Demographic characteristics and viral investigation of five patients infected with H275Y mutant viruses in 2016

Cases			Case 1	Case 2	Case 3	Case 4	Case 5
Patient information	Age (years)		75	41	37	1	2
	Sex		Female	Male	Female	Female	Female
	Area in Japan		Niigata	Nagasaki	Nagasaki	Niigata	Niigata
	Date of fever onset		25-Jan	9-Jan	10-Jan	5-Feb	12-Feb
	Date of therapy start		25-Jan	11-Jan	11-Jan	6-Feb	12-Feb
	NAIs used		Peramivir	Oseltamivir	Peramivir	Oseltamivir	Oseltamivir
	Fever duration(days)		1	1	3	3	2
	Travel history		No	No	Yes^a^	No	No
	History of NAI treatment		Yes^b^ Oseltamivir	No	No	No	No
	Underlying condition		Yes^c^	No	No	No	No
	Hospitalization		Yes^c^	No	No	No	No
Viral investigations	Date of sample collection		25-Jan	11-Jan	11-Jan	6-Feb	12-Feb
	Strain name		A/Niigata/15NU001/2016	A/Nagasaki/15N002/2016	A/Nagasaki/15N005/2016	A/Niigata/15F255/2016	A/Niigata/15F341/2016
	Neuraminidase inhibition assay (IC_50_) [nM]^d^	Oseltamivir	353.38 ± 13.32	321.59 ± 19.81	352.59 ± 23.03	320.38 ± 50.33	346.31 ± 44.37
		Peramivir	19.67 ± 2.82	23.88 ± 1.72	22.30 ± 0.83	20.10 ± 1.68	18.77 ± 0.70
		Zanamivir	0.74 ± 0.03	0.74 ± 0.04	0.69 ± 0.07	0.79 ± 0.042	0.69 ± 0.08
		Laninamivir	0.83 ± 0.03	0.74 ± 0.02	0.75 ± 0.01	0.94 ± 0.13	0.84 ± 0.07
	NA enzyme kinetics parameter^e^	Km [μM]	33.42 ± 5.15	73.22 ± 27.65	61.79 ± 12.10	29.48 ± 4.78	27.04 ± 3.02
		Vmax[μM/min]	0.33 ± 0.02	0.56 ± 0.31	0.40 ± 0.03	0.45 ± 0.06	0.31 ± 0.02

^a^Case 3 was in Fukuoka prefecture, Japan two days before the onset of fever

^b^A roommate had influenza A onset on Jan 21 and Case 1 started prophylaxis by oseltamivir

^c^Case 1 had been hospitalized for treatment for multiple myeloma

^d^Data are mean + SD of 3 independent determinations. IC_50_ values for reference sensitive virus (A/PERTH/265/2009) against four neuraminidase inhibitors (oseltamivir, peramivir, zanamivir, and laninamivir) were 1.33 ± 0.07, 0.08 ± 0.01, 0.59 ± 0.10, 0.27 ± 0.02, nM, and those for the resistant reference virus with H275Y mutation (A/PERTH/261/2009) were 276.00 ± 39.61, 30.16 ± 2.14, 0.62 ± 0.06, 0.38 ± 0.03 nM, respectively

^e^Data are mean ± SD of 3–5 independent determinations. Average values for Km [μM] and Vmax [μM/min] of 5 sensitive viruses possessing similar genetic sequences during the same seasons were 23.28 ± 2.04, and 0.36 ± 0.04. Those for the reference WHO sensitive virus (A/PERTH/265/2009) were 18.28 1.67, and 0.22 ± 0.01, and for the WHO resistant virus with H275Y mutation (A/PERTH/261/2009) were 69.38 ± 29.68, and 0.38 ±  0.05, respectively

Cases 2 to 5 visited outpatient clinics in Niigata or Nagasaki during January–February, 2016, because they had ILI (Table 1). The patients were prescribed either oseltamivir or peramivir according to the Japanese guidelines for treatment of influenza [4]. A nasopharyngeal swab was obtained from each patient prior to starting the treatment and within 48 h from their fever onset. Their fever went below 37.5 °C within 1–3 days after initiating treatment. The four cases were not epidemiologically linked. Case 3 had a history of train travel to neighboring area 2 days before the onset of symptoms.

A fluorescent-based enzyme inhibition assay using 2′-(4-methylumbelliferyl)-α-D-N-acetylneuraminic acid (MUNANA; Sigma-Aldrich Co. LLC, MO, USA) was performed to assess the susceptibility of the five H275Y A(H1N1)pdm09 viruses to four NAIs [11]. According to the World Health Organization, susceptibility of a type A virus is defined highly reduced to a specific NAIs when its IC_50_ increases by more than 100-fold compared to that of the reference susceptible virus [1, 12]. The H275Y mutant viruses showed highly reduced susceptibility indicated by a 300-fold increase in the IC_50_ values for oseltamivir and peramivir compared to the susceptible reference virus and thus assessed as resistant (Table 1). These isolates were susceptible to zanamivir and laninamivir (Table 1).

**Fig. 1:**
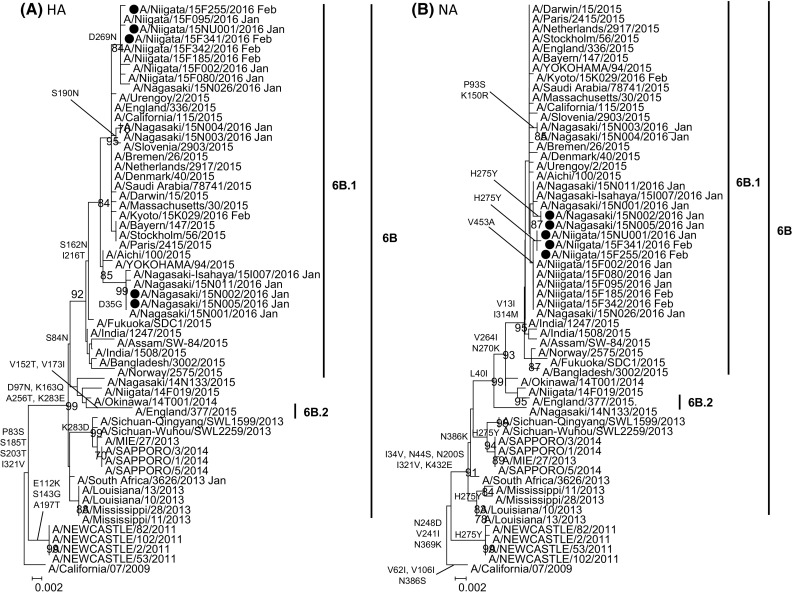
Phylogenetic analysis of the hemagglutinin (**a**) and neuraminidase gene (**b**) of the five H275Y mutant influenza A(H1N1)pdm09 strains isolated in Niigata and Nagasaki, Japan in January to February 2016. Multiple alignment was constructed by the neighbor-joining method with MEGA, version6. Bootstrap value determined for 1000 interactions. Only values of greater than 70 % are shown. The H275Y mutant strains are shown in *red*, and those detected in the 2015/2016 season are indicated in closed circle. The sensitive strains that were used for enzymatic property measurement (Km and Vmax) are colored *blue*. Reference sequences of A(H1N1)pdm09 strains downloaded from the GenBank and GISAID EpiFlu Database (www.gisaid.org.). The amino acid substitutions relative to the A/California/07/2009 strain are shown in the phylogenetic tree (Color figure online)

We next determined the hemagglutinin (HA) and NA gene sequences of the five H275Y isolates in addition to eight wild-type (H275) strains. Sanger sequencing of the HA and NA gene and phylogenetic tree analysis were performed as previously described [8]. The nucleotide sequences of HA and NA sequenced in this study are available through the Global Initiative on Sharing Avian Influenza Data (GISAID) under the accession numbers: EPI702620, EPI702622, EPI704037-EPI704042, EPI704044-EPI704049, EPI712173, EPI712174, EPI712177-EPI712184, EPI712577-EPI712580, EPI781479- EPI781486. The five H275Y isolates belonged to clade 6B.1 in both the HA and NA phylogenies forming two geographically segregated subclusters along with oseltamivir-susceptible isolates from the same season. The Niigata subcluster was defined by a D269 N amino acid substitution in the HA protein and the Nagasaki subcluster had a D35G mutation. Viruses from Niigata, but not from Nagasaki, uniquely possessed a V453A substitution in the NA protein. This suggests that the Niigata and Nagasaki H275Y viruses have evolved independently rather than being sourced from a single outbreak of resistant viruses (Fig. 1, Supplementary Table). The resistant viruses in this study commonly possessed the V241I, N369 K, and N386 K substitutions in the NA that have been previously reported among A(H1N1)pdm09 [5, 7]. Two of the substitutions, V241I and N369 K, were reported to confer robust viral fitness on the H275Y mutant viruses [5, 7, 13, 14]. These mutations are common to clades 5, 6A, 6B, and 7 strains (both susceptible and resistant) and have been reported since 2010–2011 season [5, 7, 13]. Takashita et al. have recently suggested using structural modeling that the N386 K substitution which was detected in this study slightly impairs the stabilizing effect of the V241I and N369 K mutations [7]. A survey of the influenza sequences in the public database (Influenza Sequence Database, http://www.ncbi.nlm.nih.gov/genomes/FLU/FLU.html) revealed that the N386 K mutation is not unique to H275Y viruses. We found that this mutation has been also reported among recent A(H1N1)pdm09 viruses (including H275 and H275Y strains) at increasing frequencies: 5.7 % (42/734) in 2013, 61.2 % (281/459) in 2014, 100 % (350/350) in 2015 (as of Aug 12, 2016). The effect of these mutations on the fitness of resistant viruses remains to be explored.

To assess the enzymatic properties of the resistant viruses, NA kinetic parameters (Km and Vmax) were measured for the five resistant and five sensitive isolates with genetically similar background (A/Niigata/15F185/2016, A/Kyoto/15K029/2016, A/Nagasaki-Isahaya/15I007/2016, A/Nagasaki/15N011/2016, A/Nagasaki/15N026/2016) (Fig. 1.). In addition, the WHO sensitive and resistant virus control, A/Perth/265/2009 and A/Perth/261/2009, were included in the analysis. The enzymatic kinetics was measured by a fluorescence-based assay using MUNANA and measurement of subsequent release of the fluorescent product 4-methylumbelliferone (4-MU) by NA activity, using the method reported by Marathe et al. [15]. The Km and Vmax were determined by fitting the data to Michaelis–Menten equation [15, 16] using Microsoft Excel software (Microsoft Corporation, WA, USA). Two of the viruses (A/Nagasaki/15N002/2016 and A/Nagasaki/15N005/2016) demonstrated a ~threefold increase in Km values compared to sensitive viruses, which also similar to the WHO resistant control. The remaining resistant viruses (A/Niigata/15NU001/2016, A/Niigata/15F255/2016, A/Niigata/15F341/2016) had only 1.1- to 1.5-fold increase in their Km compared to the sensitive viruses. These results suggested that the H275Y viruses possess lower affinity to the substrate which may attenuate their transmission fitness [16] as in the case of the resistant viruses reported with low frequency in the community in 2009 [2, 3]. However, the rest of viruses displayed equal or slightly higher Km values and are thus expected to have a comparable fitness to sensitive viruses, which might pose concerns about their potential to spread in the community.

Of note, one patient had received prophylactic treatment by oseltamivir because she shared the same room at the hospital with an influenza-infected patient who was also being administered oseltamivir. However, we could not obtain a sample from the roommate to confirm whether the H275Y mutation had emerged in the patient or was transmitted from the roommate (hospital-acquired). On the other hand, the remaining four patients did not have a history of NAI treatment and were not epidemiologically linked. Thus, it is likely that these strains were transmitted in the community.

In our study, three of the patients were treated with oseltamivir and two with peramivir. All patients started the treatment within 48 h of fever onset and fully recovered without complications. The duration of fever among the five cases was 1–3 days, in range with the data reported on patients infected with the H275Y mutant virus during the 2013–2014 season in Hokkaido, Japan [17]. Kakuya et al. demonstrated in a small number of pediatric patients that oseltamivir and peramivir retain the clinical effectiveness against the H275Y mutant virus [17]. In contrast, we have previously shown that children less than 6 years of age infected with the seasonal H275Y A(H1N1) virus had delayed fever resolution compared with those infected with the sensitive virus [18]. Another Japanese group demonstrated that oseltamivir was less effective for children but it was effective for adult infected with seasonal H275Y A(H1N1) virus [19]. This discrepancy in findings could be due to differences in sample size, and between seasonal A(H1N1) or A(H1N1)pdm09 infections. Further studies are warranted to better assess clinical effectiveness of oseltamivir and peramivir against the H275Y A(H1N1)pdm09 variant.

The identification of community- and potentially hospital-acquired cases of oseltamivir- and peramivir-resistant isolates is a reason for concern and highlights the importance of continued monitoring of the susceptibilities of influenza viruses to NAIs.

## Electronic supplementary material

Below is the link to the electronic supplementary material.

Supplementary material 1 (XLSX 13 kb)
